# Species Distribution Pattern and Their Contribution in Plant Community Assembly in Response to Ecological Gradients of the Ecotonal Zone in the Himalayan Region

**DOI:** 10.3390/plants10112372

**Published:** 2021-11-04

**Authors:** Inayat Ur Rahman, Aftab Afzal, Zafar Iqbal, Abeer Hashem, Al-Bandari Fahad Al-Arjani, Abdulaziz A. Alqarawi, Elsayed Fathi Abd_Allah, Mohnad Abdalla, Eduardo Soares Calixto, Shazia Sakhi, Niaz Ali, Rainer W. Bussmann

**Affiliations:** 1 Department of Botany, Hazara University, Mansehra 21300, Khyber Pakhtunkhwa, Pakistan; zafariqbal@hu.edu.pk (Z.I.); niazalitk25@gmail.com (N.A.); 2 William L. Brown Center, Missouri Botanical Garden, P.O. Box 299, St. Louis, MO 63166, USA; 3 Botany and Microbiology Department, College of Science, King Saud University, Riyadh 11451, Saudi Arabia; habeer@ksu.edu.sa (A.H.); aalarjani@ksu.edu.sa (A.-B.F.A.-A.); 4 Department of Plant Production, College of Food and Agriculture Science, King Saud University, Riyadh 11451, Saudi Arabia; alqarawi@ksu.edu.sa (A.A.A.); eabdallah@ksu.edu.sa (E.F.A.); 5 Key Laboratory of Chemical Biology (Ministry of Education), Department of Pharmaceutics, School of Phar Maceutical Sciences, Cheeloo College of Medicine, Shandong University, 44 Cultural West Road, Jinan 250012, China; mohnadabdalla200@gmail.com; 6 Entomology and Nematology Department, University of Florida, Gainesville, FL 32611, USA; calixtos.edu@gmail.com; 7 Department of Biology, University of Missouri, St. Louis, MO 63166, USA; 8 Center of Plant Sciences and Biodiversity, University of Swat, Swat 19200, Khyber Pakhtunkhwa, Pakistan; shaziasakhi@gmail.com; 9 Department of Ethnobotany, Institute of Botany, Ilia State University, 1 Botanical Street, Tbilisi 0105, Georgia; rainer.bussmann@iliauni.edu.ge

**Keywords:** transition zone, oak forest, community assembly, environmental gradients, vegetation analysis, Himalayas

## Abstract

The ecotonal zones support populations that are acclimated to changing, fluctuating, and unstable conditions, and as a result, these populations are better equipped to adjust to expected change. In this context, a hypothesis was tested that there must be vegetation dominated by unique indicator plant species under the influence of ecological gradients in the ecotonal zone of Manoor Valley (northwestern Himalaya), Pakistan. Keeping the aforementioned hypothesis in mind, detailed field studies were conducted during different seasons in 2015-18. Line transect sampling and phytosociological characteristics (density, frequency, cover, and their relative values and Importance Value) were implemented as ecological methods. This investigation documented 97 plant species recorded from seven sampling sites. The community distribution modelling revealed that the ecological variables separate the seven sampling sites into two major plant communities (*Indigofera-Parrotiopsis-Bistorta* and *Ziziphus-Leptopus-Quercus*) recognized by TWINSPAN. The IBP communities showed a positive and significant correlation with altitude (1789.6–1896.3 m), sandy soil texture with a slightly acidic pH (6.4–6.5), and higher phosphorous (9–13 mg kg^−1^). In contrast with this, the ZLQ community was recognized on the southern slope under the strong influence of high electrical conductivity (2.82–5.4 dsm^−1^), organic matter (1.08–1.25%), calcium carbonate (5.8–7.6 mg kg^−1^), potassium (202–220 mg kg^−1^), and temperature (28.8–31.8 °C). Hence, both communities were found on opposite axes with clear differences based on the ecological gradients. NMDS clustered different species with similar habitats and different stands with common species, showing that plant species and stands were in a linear combination with ecological gradients. The IPB community has the maximum number of plant species (87 species), Shannon value (H’ = 4), Simpson value (0.98), and Pielou’s evenness value (0.96). Thus, the multivariate approaches revealed unique vegetation with sharp boundaries between communities which might be due to abrupt environmental changes.

## 1. Introduction

The term “ecotone” was first coined and used for the transition zone by Frederic E. Clements in 1905 [1] and is considered as the basic unit of landscape ecology [2], having extremely unique vegetation [3], sensitive ecosystems, and sharp plant community boundaries [4], due to abrupt environmental changes [5]. The natural layering of ecosystems that occurs at different elevations owing to differing environmental conditions is referred to as altitudinal zonation in mountainous locations [6]. Nonetheless, a wide range of environmental variables, ranging from direct effects of temperature and precipitation to indirect characteristics of the mountain itself, as well as biological interactions among species, determine the boundaries of the altitudinal zones found on mountains, resulting in discrete plant communities [7,8,9]. They are natural assemblages of co-evolved individual populations, which form visible units [10,11]. Ecotones often appear along ecological gradients [12]. Such gradients are created because of spatial shifts in elevation, climate, soil, and many other ecological factors [13]. These regions are sometimes regarded as dynamic zones of community interaction that are inherently unstable over time [14]. Ecotones, as indicated by Odum [15], are more than just a boundary or an edge; the idea of an ecotone presupposes the presence of active interaction between two or more ecosystems, with features that are not present in either of the neighboring ecosystems.

Ecotones have been investigated from an ecological perspective for the past four decades [13,16,17,18] and have recently received considerable attention in the context of biodiversity protection. Ecotones are “natural laboratories” where researchers may study a range of evolutionary processes, such as speciation and the emergence of new species. It is greatly influenced by various environmental gradients, i.e., edaphic, climatic, and physiographic variables [19]. In those regions, environmental conditions that fluctuate over time and space might be useful indicators of environmental change and ecological responses to climate change. Some researchers claim that ecotonal zones support populations that are acclimated to changing, fluctuating, and unstable conditions, and that as a result, these populations are better equipped to adjust to expected change. Ecotones’ efficacy as early predictors of climatic changes and the ways that ecological groups and systems adapt to change might be a promising future endeavor. Ecotones can support distinct species or indicators that are less common or do not exist elsewhere. Therefore, a hypothesis was tested that there must be vegetation dominated by unique indicator plant species in the ecotonal zone. The current study was planned to determine the species distribution pattern, species contribution, and major plant community assembly in association with the ecological gradients of the said zone.

## 2. Materials and Methods

### 2.1. Study Area

**Figure 1:**
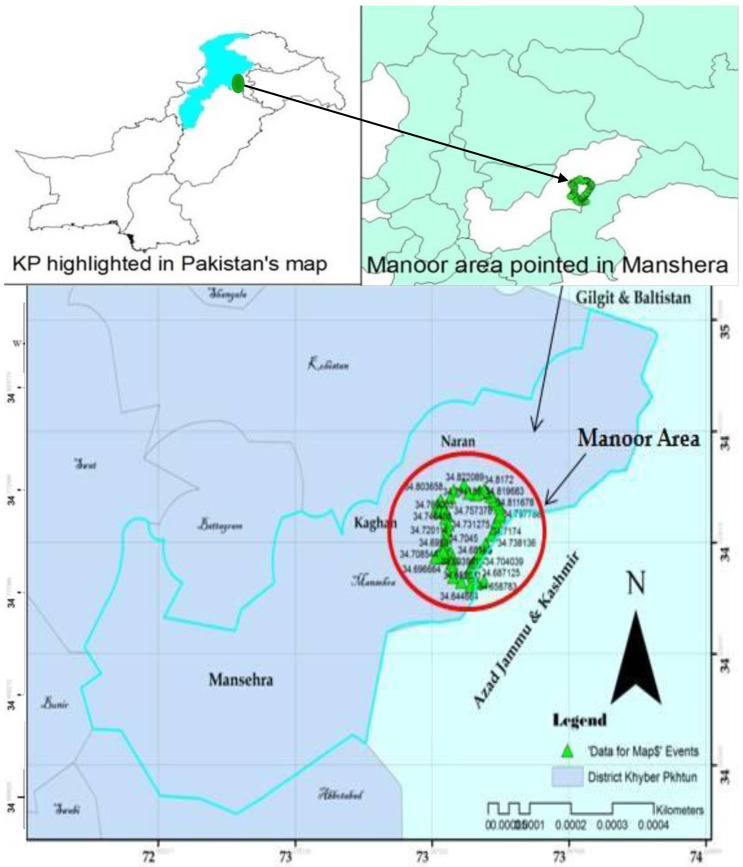
Map of the study area generated through ArcGIS.

The current project lies in the ecotonal zone of the Manoor Valley. Manoor Valley is reached from the main Kaghan Valley road at the junction ‘Mahandri’ [20] and is about 50 km north of Balakot [21,22]. Geographically, Manoor Valley (Figure 1) is situated in northwestern Pakistan (34.68165 N to 34.83869 N latitude and 73.57520 E to 73.73182 E longitude) as a part of the mountainous series collectively known as the Himalayas [23]. The entire area is formed by crosswise ridges of mountains on either side of the Manoor river, which flows in a northeast to southwest direction down the valley emerged from Malika Parbat (‘Queen of Mountains’, elevation 5279 m).

### 2.2. Vegetation Sampling

Detailed field visits were conducted to the ecotonal/transition zone of Manoor Valley, Himalaya, Pakistan. Line transect ecological technique was used for vegetation sampling [24,25,26,27,28,29]. The study area was subdivided into seven sampling sites, and three transects each of 50 m at each sampling site were randomly selected and sampled [30]. The interval distance between the transects were 100 m and 200 m interval between each sampling site. The individuals of plant species falling precisely on the line were recorded. The phytosociological parameters such as density, frequency, cover, and their relative values and Importance Value (IV) were calculated following the methodology of Curtis and McIntosh [31] and Buckland et al. [25]. Plant specimens were collected, serially tagged with a field number, pressed between the blotting papers for drying, poisoned, and mounted on standard herbarium sheets following the recommended procedures [32,33]. All the specimens were identified with the help of Flora of Pakistan and other available literature [34,35,36]. Afterwards, the plant specimens were deposited in the Herbarium of Hazara University, Mansehra, Pakistan for accession numbers.

### 2.3. Ecological Gradients

GPS locations were recorded for each sampling site. Aspect of the mountain, i.e., east (E), west (W), south (S), and north (N), was determined with the help of clinometer, and latitude, longitude, and altitude using geographical positioning system (GPS). Regarding edaphology, soil samples of 200–400 g were collected from three random points within each sampling site from depth of 0–30 cm [37] and mixed thoroughly to make a composite sample [38]. Samples were placed in polythene bags and properly tagged with permanent marker. Moreover, rocks and other raw materials were removed by sieving and then the remaining samples were shade dried. Physicochemical analyses, i.e., soil texture (clay, sand, silt, loam), pH [39], electrical conductivity (EC) [40], organic matters (OM) [41], nitrogen (N), phosphorus (P), potassium (K), and calcium carbonate (CaCO_3_) concentrations were determined for each soil sample [42,43,44]. Moreover, other climatic gradients (i.e., barometric pressure, dew point, humidity, heat index, temperature, wet bulb, and wind speed) were determined with aid of handheld weather station (Kestrel weather tracker 4000).

### 2.4. Data Analyses

All the data of plant species and ecological gradients recorded in the field were transferred to the MS-Excel spreadsheets and arranged to determine the relationship among them [45,46]. The analyses were conducted using matrices of IV data from all of the recorded plant species (97 species) and ecological gradients from seven sampling sites (Tables S1 and S2) using two separate spreadsheets like IV of species x sampling sites and ecological gradients data x sampling sites [19,47]. A georeferenced map was prepared using ArcGIS version 10.1 to show the study area. For multivariate ordination analyses, CANOCO 5 [45], PC-ORD [48], and R 3.6.1 [49] were used. Species area curve (SAC) was drawn to evaluate the adequacy level of area sampled in relation to the vegetation [19]. Cluster analysis (CA), two-way cluster analysis (TWCA) [50] and two way indicator species analysis (TWINSPAN) were carried out using PC-ORD software [51] for the identification of major groups [19]. Moreover, based on species categorization and sample clustering [52], TWINSPAN was processed for the recognition of the plant communities/indicator species [51].

Non-multidimensional scaling ordination (NMDS) and Principal Component Analysis (PCA) were done to visualize the floristic associations among the main taxonomic units (communities) [53] using the package “vegan” [54]. Analysis of Similarities (ANOSIM *p* < 0.001, 5039 permutations) was carried out to determine the significant variations in recorded plant species composition between communities. The ANOSIM was processed in R 3.5.1 software using the package “vegan” [54]. To compare the parameters (diversity and ecological) evaluated among the two communities, we conducted a GLM with Gaussian error distribution (except for species richness, in which we used Poisson distribution) followed by Likelihood ratio test using the ‘stats’ and ‘car’ [55] packages, respectively. In addition, we measured Pielou’s evenness, Shannon, and Simpson diversity indices for each stand of each plant community using Gaussian error [19,56].

## 3. Results

A very narrow range of Himalayan Subtropical-Temperate Ecotone was observed mostly on the southern slopes and foothills of the Manoor river. In total, 97 species were recorded from seven stands.

### 3.1. Vegetation Characterization

**Figure 2:**
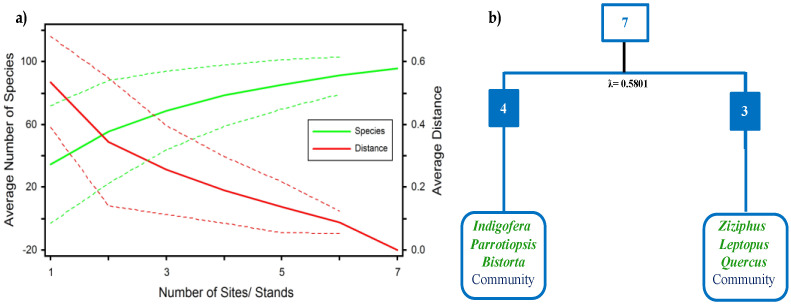
Compilation of records of plant diversity along the ecological gradients in the Himalayan Subtropical-Temperate Ecotone/Evergreen Oak Forest of the study area: (**a**) The species area curves of 97 plant species distributed among seven sampling sites; (**b**) TWINSPAN classification of the Himalayan Subtropical-Temperate Ecotonal zone of Manoor Valley.

**Figure 3:**
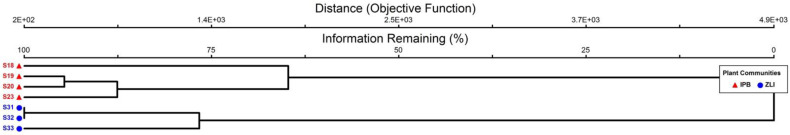
Cluster analysis indicating two different plant communities’ recognition based upon the grouping of seven sampling sites and 97 plant species. IPB: *Indigofera-Parrotiopsis-Bistorta* and ZLQ: *Ziziphus-Letopus-Quercus*.

**Figure 4:**
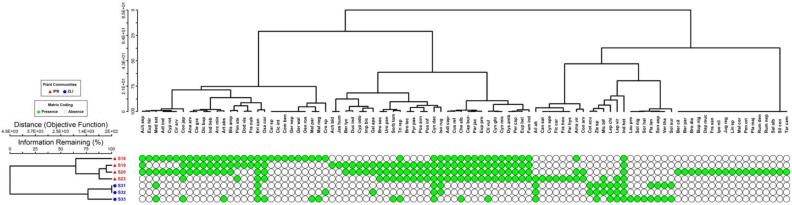
TWCA analysis indicating two different groups recognition based up on 97 plant species recorded from seven sampling sites. Each green dot shows the presence, and a white dot indicates the absence of a species within each stand or sampling site. IPB: *Indigofera-Parrotiopsis-Bistorta* and ZLQ: *Ziziphus-Letopus-Quercus*.

The species area curve (SAC) analysis showed that the maximum number of plant species appeared in the sixth stand and the species curve became parallel after that, as no new species were recorded later, which clearly shows that the sampled area was enough for the targeted vegetation (Figure 2a). According to TWINSPAN (Figure 2b), CA (Figure 3), and TWCA (Figure 4), a total of two different major plant communities (*Indigofera-Parrotiopsis-Bistorta* and *Ziziphus-Leptopus-Quercus*) were recognized by clustering all the recorded plant species (97 species) in seven stands by the influence of ecological gradients. They ranged from 1780.8 m to 1896.3 m. Two different clusters were observed by the TWINSPAN method, which showed a high cluster heterogeneity value (Lambda = 0.5801). Each community was comprised of different indicator species (i.e., *Indigofera heterantha, Parrotiopsis jacquemontiana, Bistorta amplexicaulis* and *Ziziphus* sp., *Leptopus chinensis, Quercus incana*) and was recorded at different altitudes (Figure 2b).

### 3.2. Vegetation along the Ecological Gradients

**Figure 5:**
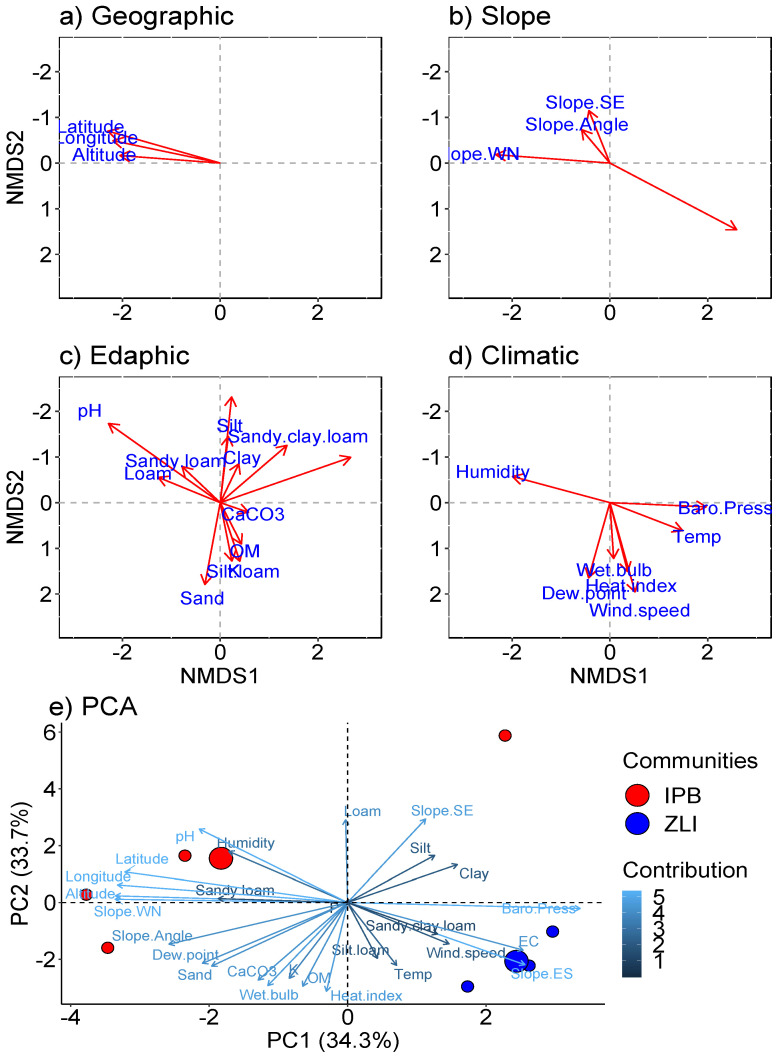
Multivariate analyses among plant communities of Himalayan Subtropical-Temperate Ecotone/Evergreen Oak Forest and ecological gradients. (**a**–**d**), Non-Multidimensional Scaling (NMDS) among vegetation communities and geographic (**a**), slope (**b**), edaphic (**c**), and climatic (**d**) gradients. (**e**) Principal Component Analysis (PCA) ordination of two plant communities along the ecological gradients. Species contribution analysis for community ordination in NMDS is depicted in Table 1. IPB: *Indigofera-Parrotiopsis-Bistorta* and ZLQ: *Ziziphus-Letopus-Quercus*.

Correlations between Himalayan Subtropical-Temperate Ecotone plant communities and ecological gradients were illustrated through NMDS (Figure 5a–d) and PCA (Figure 5e). Communities are related to specific ecological variables such as geographic, slope, edaphic, and climatic variables. Altitude, slope NE, slope N, K, pH, OM, silt, sand, clay, temperature, heat index, wind speed, and altitudinal density were the most representative ecological variables which drive the plant community structure and diversity along the altitudinal gradient. In both ordinations, the ecological variables separate the seven sampling sites into two major plant communities already demonstrated by TWINSPAN (Figure 2b). In constrained PCA ordination, the maximum explanatory variation was accounted for PC1 axis (34.3%) and lower variation on the PC2 axis (33.7%). Maximum strength was recorded for the most important ecological gradients, i.e., the profound influence of the altitudinal gradient was revealed by dividing the vegetation of the Himalayan Subtropical-Temperate Ecotone into two communities (Figure 2b–d).

The IBP communities showed a positive and significant correlation with altitude (1789.6–1896.3 m), sandy soil texture with a slightly acidic pH (6.4–6.5), and higher phosphorous (9–13 mg kg^−1^). In contrast to this, the ZLQ community was recognized on the southern slope. The edaphic gradients structuring the indicators and associated plant species of this community were high electrical conductivity (2.82–5.4 dsm^−1^), organic matter (1.08–1.25%), calcium carbonate (5.8–7.6 mg kg^−1^), and potassium (202–220 mg kg^−1^). Temperature (28.8–31.8 °C), barometric pressure (815.6–816.4 pa), and heat index (29.8–34.3) were found to have a positive and significant influence when compared to the IPB community. As a result, both communities were discovered on opposing axes, with distinct differences based on ecological gradients.

### 3.3. Analysis of Similarity (ANOSIM)

The ANOSIM was conducted to measure the species contribution for the site distribution pattern. Significant variation in plant species composition between communities was found (ANOSIM *p* < 0.001, 5039 permutations). Out of 97 species, 16 were significant and greatly contributed to the variation in plant community ordination in NMDS (Table 1).

### 3.4. Significance Testing of Plant Communities in Relation to Studied Variables

**Figure 6:**
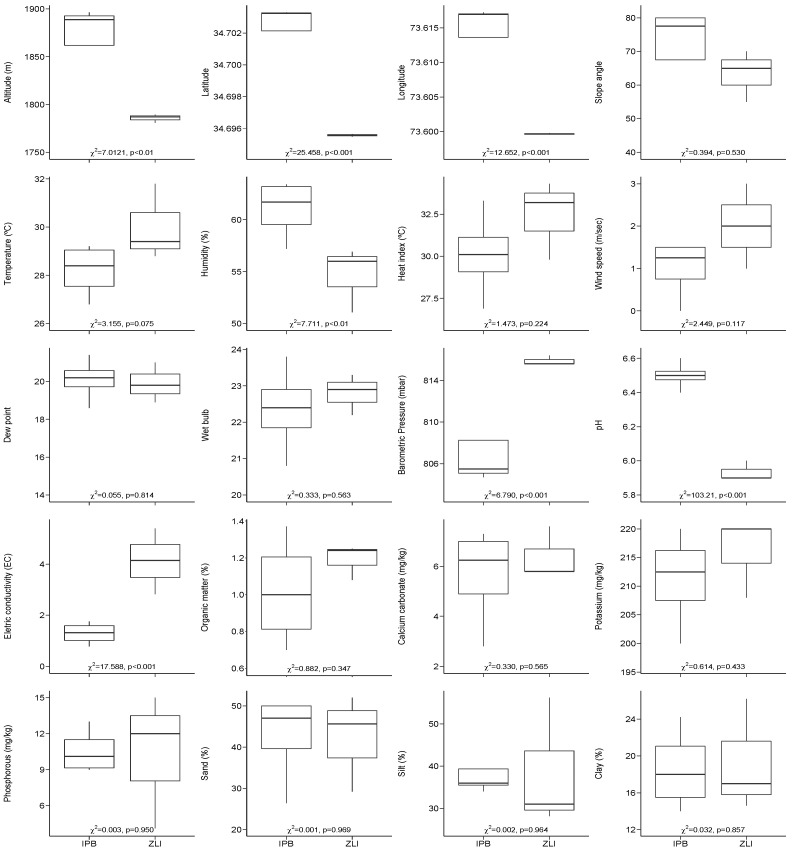
Variation of ecological parameters between the two plant communities evaluated in our study (GLM results, and associated *p*-values are displayed at each plot).

GLM analyses of the 20 studied variables distributed in different groups (geographic, slope, climatic and edaphic gradients) between two plant communities showed that most of the gradients were significantly different (*p*-value < 0.05; Figure 6). Nonetheless, slope angle, humidity, dew point, wind speed, wet bulb, organic matter, soil texture, potassium, and calcium carbonate did not show significant differences (Figure 6).

### 3.5. Species Richness and Diversity Indices

**Figure 7:**
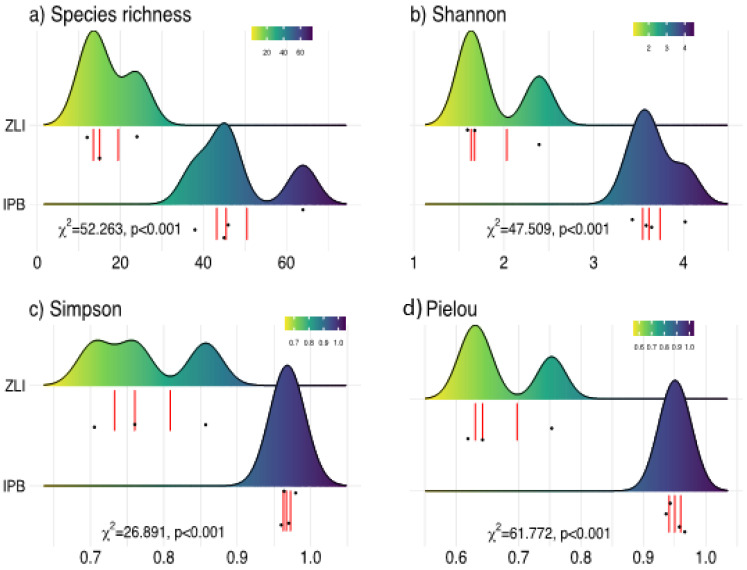
Variation of diversity indexes between the two plant communities evaluated in Himalayan Subtropical-Temperate Ecotone/Evergreen Oak Forest (GLM results, and associated *p*-values are displayed at each plot). (**a**–**d**), Relationship between species richness (**a**), Shannon diversity (**b**), Simpson diversity (**c**), and Pielou’s evenness (**d**) in relation to two plant communities based on the stand’s values. *X*-axis is displayed in ascendant altitudinal gradient.

Species richness values ranged from 30 and 87 species in two plant communities (Figure 7a). The highest number of plant species was reported in the IPB community (87 species) at 1789.6–1896.3 m altitude, followed by ZLQ (30 species) at the altitudinal range of 1780.8–1789.3m. The Shannon Diversity index values ranged between 2.4 and 4 (Figure 7b). The IPB community had the maximum value (H’ = 4), followed by the ZLQ community (2.4). The Simpson’s dominance index values ranged between 0.86–0.98. The IPB community had the maximum value (0.98) between the recorded communities. Moreover, the lowest Simpson dominance value has been calculated for the ZLQ community (Figure 7c). The Pielou’s evenness index values ranged between 0.75 and 0.96 (Figure 7d). The IPB community had the maximum value (0.96); on the other hand, the ZLQ community had the lowest Pielous’s evenness value.

## 4. Discussion

Mountains are the most remarkable land forms on the earth‘s surface with major vegetation zones based upon environmental variations [57]. Ecotones commonly coincide with areas of abrupt climatic transition along ecological gradients [16]. They can be found at a variety of spatial dimensions [58], ranging from continental-scale biome transitions to small-scale ecotones where plant communities and microhabitats coincide [12]. A very narrow range of Himalayan Subtropical-Temperate Ecotone was observed mostly on the southern slopes and foothills of the Manoor river. Aspect encourages habitat heterogeneity and induces micro-environmental variation in vegetation patterns [59,60]. The community distribution pattern (Figure 5a–e) revealed that ecological variables divided the seven sampling sites and their 97 native species into two major plant communities, identified by CA, TWCA, and TWINSPAN classifications. Ordination techniques have been widely used to reveal plant species diversity, distribution, and community recognition along ecological gradients [61,62]. Furthermore, multivariate statistical approaches can assist ecologists effectively in describing and elaborating the structure of vegetation, as well as quantifying the effects of ecological gradients on vegetation and/or a group of species [63,64]. Statistical tools can provide an efficient means of reducing the complexity inherent in natural vegetation and of detecting important environmental factors that explain this complexity [48,65].

*Indigofera-Parrotiopsis-Bistorta* and *Ziziphus-Leptopus-Quercus* were identified as the major plant communities (ranges from 1780.8 m to 1896.3m) by grouping all the recorded plant species (97 species) and the seven stands were strongly influenced by ecological gradients. Plant communities that can be described both physiognomically and floristically have a well-defined structure in relation to abiotic and biotic factors [31,66,67]. Using more or less the same techniques, a similar pattern of species distribution was reported from another mountainous area [68]. The evergreen *Quercus incana* has also been reported as an indicator species in the subtropical-temperate ecotonal zone of the Nandiar catchment, Batagram [69], as have *Bisorta amplexicaule* and *Indigofera heterantha* at a lower xeric elevation in the ecotonal range of District Khurram, Pakistan [18]. Such similarities clearly reveals that some abiotic factors have the influence on the distribution of vegetation [19]. Our results revealed that the IBP community had been strongly influenced by altitude (1789.6–1896.3 m), sandy soil texture with slightly acidic pH (6.4–6.5) and higher phosphorous (9–13 mg kg^−1^). In contrast with this, the ZLQ community was recognized on the southern slope under the strong influence of high electrical conductivity (2.82–5.4 dsm^−1^), organic matter (1.08–1.25%), calcium carbonate (5.8–7.6 mg kg^−1^), potassium (202–220 mg kg^−1^), and temperature (28.8–31.8 °C). Thus, both the communities were clustered with different leading indicator species, and similar habitats and different stands with common species, showing that plant species and stands were in a linear combination with ecological gradients. Similarly, many other researchers [70] stated that aspect, altitude, and soil depth are the most influential ecological variables for determining the community structure in the Naran Valley.

The highest number of plant species were reported in the IPB community (87 species) at 1789.6–1896.3 m altitude. The IPB community had the maximum Shannon value (H’ = 4), Simpson value (0.98), and Pielou’s evenness value (0.96). The distribution of species richness along the altitudinal gradient is governed by series of interacting biological and climatic factors [71,72,73]. The gradual decrease in species richness along with increasing altitude is considered as a general pattern [74]. In such studies, species richness [75,76] and abundances tend to peak in ecotonal regions, according to several investigations, however outliers exist. Plant species are distributed in different type of habitats; but in their own territory, they usually show an abundance that represents their special ecological optimum [57]. Due to this reason, the composition of distinct units is regarded as a function of changing habitat conditions along ecological gradients [77].

## 5. Conclusions

The current study provides the baseline and first insights into spatial distribution and vegetation mapping in response to ecological gradients of the ecotonal zone in Manoor Valley, Himalaya, Pakistan. The community distribution pattern revealed that the ecological variables separate the seven sampling sites into two major plant communities. Both the communities were clustered with different leading indicator species, sharing a similar habitat and different stands with common species, showing that plant species and stands were in a linear combination with ecological gradients. Significant variation in plant species composition between communities was recorded (ANOSIM *p* < 0.001, 5039 permutations). Out of 97 species, 16 were found significant that greatly contributed to the variation in plant community ordination. Hence, the multivariate approaches revealed unique vegetation with sharp boundaries between communities, which might be due to abrupt environmental changes. In the context of global change, including land-use and climate change, research on the influence of ecotones on biodiversity is an essential future direction. Since climate change is projected to be quick and intense in ecosystem border zones [78] and these regions might possibly act as “early warning” indicators of global changes by studying variations in ecotone locations over time [79,80]. The response, however, is dependent on the geographical and temporal scales studied, and it may be a helpful indication primarily at global spatial scales and on fairly coarse durations.

## Figures and Tables

**Table 1:** Species contribution analysis for the site distribution pattern, referred to as intrinsic variables. Significant species are displayed in bold.

Species	Abbreviations	NMDS1	NMDS2	r2	Pr(>r)
*Achyranthes aspera* L.	Ach.asp	−0.96987	0.24364	0.4507	0.257
*Achyranthes bidentata* Blume	Ach.bid	−0.70128	−0.71289	0.1373	1.000
***Adiantum capillus-veneris* L.**	**Adi.cap−ven**	**−0.96337**	**−0.26818**	**0.8058**	**0.039**
*Aesculus indica* (Wall. ex Camb.) Hook.	Adi.ind	−0.91470	0.40413	0.3475	0.372
*Ailanthus altissima* (Mill.) Swingle	Ail.alt	−0.07487	0.99719	0.0154	0.968
*Amaranthus viridis* L.	Ama.vir	−0.78624	−0.61792	0.4079	0.356
*Anagallis arvensis* L.	Ana.arv	−0.99174	0.12829	0.5186	0.242
*Arctium minus* (Hill) Benh.	Arc.min	−0.99793	0.06433	0.5744	0.187
*Artemisia absinthium* L.	Art.abs	−0.76707	0.64156	0.6196	0.155
*Bergenia ciliata* (Haw.) Sternb.	Ber.cil	−0.93351	−0.35856	0.1997	0.558
*Berberis lycium* Royle	Ber.lyc	−0.86966	−0.49365	0.3704	0.280
*Berberis parkeriana* C.K. Schneid.	Ber.par	−0.93351	−0.35856	0.1997	0.558
*Bistorta amplexicaulis* (D. Don) Greene	Bis.amp	−0.91497	0.40353	0.3037	0.434
*Bromus diandrus* Roth.	Bro.dia	−0.93351	−0.35856	0.1997	0.558
*Bromus secalinus* L.	Bro.sec	−0.69951	−0.71463	0.6587	0.078
*Bromus tectorum* L.	Bro.tec	−0.66386	−0.74786	0.6800	0.080
*Bupleurum nigrescens* E. Nasir	Bup.nig	−0.93351	−0.35856	0.1997	0.558
*Cannabis sativa* L.	Can.sat	−0.35050	−0.93656	0.1671	0.701
*Capsella bursa-pastoris* (L.) Medik.	Cap.bur−pas	−0.91592	−0.40136	0.7852	0.067
*Carex* sp.	Car.sp	−0.61623	0.78757	0.1488	0.859
*Chenopodium album* L.	Che.alb	−0.99837	−0.05713	0.5975	0.160
*Cichorium intybus* L.	Cic.int	−0.61623	0.78757	0.1488	0.859
*Cirsium arvense* (L.) Scop.	Cir.arv	−0.89265	0.45075	0.3329	0.437
*Clematis grata* Wall.	Cle.gra	−0.98884	0.14896	0.4495	0.313
***Clinopodium vulgare* L.**	**Cli.vul**	**−0.95549**	**−0.29502**	**0.8765**	**0.025**
*Commelina benghalensis* L.	Com.ben	−0.61623	0.78757	0.1488	0.859
*Convolvulus arvensis* L.	Con.arv	−0.54123	−0.84088	0.3380	0.380
*Conyza japonica* (Thunb.) Less. ex Less.	Con.jap	−0.82955	0.55844	0.5936	0.154
***Cotoneaster acuminatus* Wall. ex Lindl.**	**Cot.acu**	**0.69116**	**−0.72270**	**0.9339**	**0.031**
*Crotalaria* sp.	Cro.sp	0.81696	−0.57670	0.0080	0.970
*Cuscuta reflexa* Roxb.	Cus.ref	−0.97647	−0.21564	0.6624	0.118
*Cynoglossum apenninum* L.	Cyn.ape	−0.35050	−0.93656	0.1671	0.701
*Cynodon dactylon* (L.) Pers.	Cyn.dac	−0.99873	0.05040	0.5807	0.180
*Cynoglossum glochidiatum* Wall. ex Benth.	Cyn.glo	−0.84752	−0.53076	0.7874	0.064
***Cynoglossum microglochin* Benth.**	**Cyn.mic**	**−0.84104**	**−0.54098**	**0.8332**	**0.040**
*Cyperus odoratus* L.	Cyp.odo	−0.79409	−0.60781	0.3201	0.430
*Cyperus rotundus* L.	Cyp.rot	−0.90457	0.42633	0.3411	0.397
*Daphne mucronata* Royle	Dap.muc	−0.93351	−0.35856	0.1997	0.558
*Dicliptera bupleuroides* Nees	Dic.bup	−0.96871	0.24818	0.4817	0.265
*Dodonaea viscosa* (L.) Jacq.	Dod.vis	−0.99805	−0.06247	0.2555	0.583
*Duchesnea indica* (Andx) Fake.	Duc.ind	−0.84188	−0.53966	0.3961	0.256
***Dysphania ambrosioides* (L.) Mosyakin & Clemants**	**Dys.amb**	**−0.90846**	**−0.41798**	**0.9613**	**0.013**
*Erigeron canadensis* L.	Eri.can	−0.21391	0.97685	0.5741	0.173
***Euphorbia helioscopia* L.**	**Eup.hel**	**−0.93448**	**−0.35602**	**0.9337**	**0.012**
*Euphorbia hirta* L.	Eup.hir	−0.94701	0.32120	0.3630	0.364
*Euphorbia prostrata* Ait.	Eup.pro	−0.89409	−0.44789	0.6637	0.105
*Ficus carica* L.	Fic.car	−0.35050	−0.93656	0.1671	0.701
*Fraxinus hookeri* Wenz.	Fra.hoo	−0.35050	−0.93656	0.1671	0.701
*Fragaria nubicola* (Hook. f.) Lindl. ex Lacaita	Fra.nub	−0.98608	0.16625	0.2652	0.557
*Fraxinus xanthoxyloides* (G. Don) DC.	Fra.xan	−0.93351	−0.35856	0.1997	0.558
***Fumaria indica* (Hausskn) Pugsley**	**Fum.ind**	**−0.81941**	**−0.57321**	**0.9588**	**0.007**
*Galium aparine* L.	Gal.apa	−0.41051	−0.91186	0.2183	0.625
*Geranium nepalense* Sweet.	Ger.nep	−0.61623	0.78757	0.1488	0.859
*Geranium wallichianum* D. Don ex Sweet	Ger.wal	−0.61623	0.78757	0.1488	0.859
*Impatiens bicolor* Royle.	Imp.bic	−0.79600	−0.60529	0.3248	0.430
*Indigofera hebepetala* Baker	Ind.heb	−0.94517	0.32659	0.4344	0.284
*Indigofera heterantha* Brandis	Ind.het	0.30346	0.95285	0.2066	0.644
*Ipomoea nil* (L.) Roth	Ipo.nil	−0.93351	−0.35856	0.1997	0.558
*Isodon rugosus* (Wall. ex Benth.) Codd	Iso.rug	−0.36383	−0.93146	0.6163	0.137
*Jasminum humile* L.	Jas.hum	−0.70128	−0.71289	0.1373	1.000
*Juglans regia* L.	Jug.reg	−0.93351	−0.35856	0.1997	0.558
*Launaea procumbens* (Roxb.) Ramayya and Rajagopal	Lau.pro	0.11700	0.99313	0.8884	0.148
***Leptopus chinensis* (Bunge) Pojark.**	**Lep.chi**	**0.94983**	**0.31278**	**0.9680**	**0.015**
*Leptodermis virgata* Edgew. ex Hook.F.	Lep.vir	0.60228	−0.79828	0.3181	0.457
*Lindelofia* sp.	Lin.sp	−0.93351	−0.35856	0.1997	0.558
*Malvastrum coromandelianum* (L.) Garcke	Mal.cor	−0.93351	−0.35856	0.1997	0.558
*Malva parviflora* L	Mal.par	−0.13602	0.99071	0.4815	0.224
*Malva neglecta* Wallr.	Mal.neg	−0.21533	0.97654	0.3408	0.295
*Medicago sativa* L.	Med.sat	−0.67170	0.74083	0.3808	0.324
***Micromeria biflora* (Ham.) Bth.**	**Mic.bif**	**0.50585**	**−0.86262**	**0.8617**	**0.044**
*Oenothera rosea* L. Her ex Aiton	Oen.ros	−0.61623	0.78757	0.1488	0.859
*Oxalis corniculata* L.	Oxa.cor	−0.70137	0.71279	0.5950	0.189
*Parthenium hysterophorus* L.	Par.hys	−0.35050	−0.93656	0.1671	0.701
*Parrotiopsis jacquemontiana* (Decne.) Rehder	Par.jac	−0.93944	−0.34272	0.6925	0.099
*Pennisetum orientale* Rich.	Pen.ori	−0.93351	−0.35856	0.1997	0.558
***Persicaria capitata* (Buch.-Ham. ex D.Don) H.Gross**	**Per.cap**	**−0.89091**	**−0.45418**	**0.9782**	**0.019**
*Pimpinella stewartii* (Dunn) Nasir	Pim.ste	−0.99881	0.04881	0.3136	0.413
***Plantago lanceolata* L.**	**Pla.lan**	**0.18423**	**0.98288**	**0.8884**	**0.045**
*Plantago major* L.	Pla.maj	−0.93351	−0.35856	0.1997	0.558
*Poa annua* L.	Poa.ann	−0.67556	−0.73730	0.5850	0.132
*Poa infirma* Kunth	Poa.inf	−0.64465	−0.76448	0.5003	0.232
*Pyrus pashia* Buch.-Ham. ex D.Don	Pyr.pas	−0.64295	−0.76591	0.6690	0.094
*Quercusincana* Bartram	**Que.inc**	**0.74983**	**0.35278**	**0.9480**	**0.035**
*Rumex dentatus* L.	Rum.den	−0.93351	−0.35856	0.1997	0.558
*Rumex nepalensis* Sprenge	Rum.nep	−0.93351	−0.35856	0.1997	0.558
*Salix alba* L.	Sal.alb	−0.93351	−0.35856	0.1997	0.558
*Silene conoidea* L.	Sil.con	−0.93351	−0.35856	0.1997	0.558
*Solanum nigrum* L.	Sol.nig	0.11700	0.99313	0.8884	0.148
Solanum surattense Burm F.	Sol.sur	0.32179	0.94681	0.7930	0.089
*Sonchus asper* (L.) Hill	**Son.asp**	**0.18595**	**0.98256**	**0.8879**	**0.045**
*Sorghum halepense* (L.) Pers.	Sor.hal	0.11700	0.99313	0.8884	0.148
*Sorbaria tomentosa* (Lindl.) Rehder	Sorb.tom	−0.99934	−0.03641	0.4449	0.309
*Taraxacum officinale* aggr. F.H. Wigg.	Tar.off	−0.93351	−0.35856	0.1997	0.558
*Trifolium repens* L.	**Tri.rep**	**−0.96524**	**−0.26135**	**0.8350**	**0.024**
*Urochloa panicoides* P. Beauv.	Uro.pan	−0.69247	−0.72145	0.6679	0.082
*Verbascum thapsus* L.	**Ver.tha**	**0.15572**	**0.98780**	**0.8935**	**0.045**
*Ziziphus* sp.	**Ziz.sp**	**0.91809**	**−0.39636**	**0.9309**	**0.014**

## Data Availability

Not applicable.

## Supplementary Materials

The following are available online at https://www.mdpi.com/article/10.3390/plants10112372/s1, Table S1. List of plant species with their Importance values (IV) calculated based on vegetative characteristics of each sampling sites. Table S2. Means of environmental variables measured at three different transects of each sampling site were recorded (wind speed averages are given as integers).

## References

[1] Clements F.E. (1905). Research Methods in Ecology.

[2] Casalini A.I., Bouza P.J., Bisigato A.J. (2019). Geomorphology, soil and vegetation patterns in an arid ecotone. Catena.

[3] Attrill M., Rundle S. (2002). Ecotone or Ecocline: Ecological Boundaries in Estuaries. Estuar. Coast. Shelf Sci..

[4] Gonçalves E.T., Souza A.F. (2014). Floristic variation in ecotonal areas: Patterns, determinants and biogeographic origins of sub-tropical forests in South America. Austral. Ecol..

[5] Arellano G., Umaña M.N., Macía M.J., Loza M.I., Fuentes A., Cala V., Jørgensen P.M. (2017). The role of niche overlap, environmental heterogeneity, landscape roughness and productivity in shaping species abundance distributions along the Amazon–Andes gradient. Glob. Ecol. Biogeogr..

[6] Frahm J.-P., Gradstein S.R. (1991). An altitudinal zonation of tropical rain forests using byrophytes. J. Biogeogr..

[7] Motiekaityte V., Motiekaityté V. (2006). Conservation Diversity of Vascular Plants and their Communities in situ, applying the Conception of Ecosystem Pool. Ekologija.

[8] Myers N., Mittermeier R.A., Mittermeier C.G., Da Fonseca G.A., Kent J. (2000). Biodiversity hotspots for conservation priorities. Nature.

[9] Shipley B., Keddy P.A. (1987). The individualistic and community-unit concepts as falsifiable hypotheses. Theory and Models in Vegetation Science.

[10] Vleminckx J., Schimann H., Decaëns T., Fichaux M., Vedel V., Jaouen G., Roy M., Lapied E., Engel J., Dourdain A. (2019). Coordinated community structure among trees, fungi and invertebrate groups in Amazonian rainforests. Sci. Rep..

[11] Hu A., Wang J., Sun H., Niu B., Si G., Wang J., Yeh C.-F., Zhu X., Lu X., Zhou J. (2020). Mountain biodiversity and ecosystem functions: Interplay between geology and contemporary environments. ISME J..

[12] Kark S. (2013). Ecotones and Ecological Gradients. Ecological Systems.

[13] Erdős L. (2011). On the terms related to spatial ecological gradients and boundaries. Acta Biol. Szeged..

[14] Kent M., Gill W.J., Weaver R.E., Armitage R.P. (1997). Landscape and plant community boundaries in biogeography. Prog. Phys. Geogr. Earth Environ..

[15] Vance R.B. (1953). Odum. Fundamentals of Ecology (Book Review). Soc. Forces.

[16] Ludwig J.A., Cornelius J.M. (1987). Locating Discontinuities along Ecological Gradients. Ecology.

[17] Delgado-Mellado N., Larriba M., Navarro P., Rigual V., Ayuso M., García J., Rodríguez F. (2018). Thermal stability of choline chloride deep eutectic solvents by TGA/FTIR-ATR analysis. J. Mol. Liq..

[18] Hussain M., Khan S.M., Abd_Allah E.F., Ul Haq Z., Alshahrani T.S., Alqarawi A.A., Ur Rahman I., Iqbal M., Abdullah–Ahmad H. (2019). Assessment of Plant communities and identification of indicator species of an ecotonal forest zone at durand line, district Kurram, Pakistan. Appl. Ecol. Environ. Res..

[19] Rahman I.U., Afzal A., Iqbal Z., Bussmann R.W., Alsamadany H., Calixto E.S., Shah G.M., Kausar R., Shah M., Ali N. (2020). Ecological gradients hosting plant communities in Himalayan subalpine pastures: Application of multivariate approaches to identify indicator species. Ecol. Inform..

[20] Rahman I.U., Afzal A., Iqbal Z., Abd Allah E.F., Alqarawi A.A., Calixto E.S., Ali N., Ijaz F., Kausar R., Alsubeie M.S. (2019). Role of multivariate approaches in floristic diversity of Manoor Valley (Himalayan Region), Pakistan. Appl. Ecol. Environ. Res..

[21] Rahman I.U., Afzal A., Iqbal Z., Ijaz F., Ali N., Asif M., Alam J., Majid A., Hart R., Bussmann R.W. (2018). First insights into the floristic diversity, biological spectra and phenology of Manoor valley, Pakistan. Pakistan J. Bot..

[22] Rahman I.U., Ijaz F., Afzal A., Iqbal Z., Ali N., Khan S.M. (2016). Contributions to the phytotherapies of digestive disorders: Traditional knowledge and cultural drivers of Manoor Valley, Northern Pakistan. J. Ethnopharmacol..

[23] Rahman I.U., Afzal A., Iqbal Z., Hart R., Allah E.F.A., Hashem A., Alsayed M.F., Ijaz F., Ali N., Shah M. (2019). Herbal teas and drinks: Folk medicine of the Manoor Valley, Lesser Himalaya, Pakistan. Plants.

[24] Buckland S.T., Anderson D.R., Burnham K.P., Laake J.L., Borchers D.L., Thomas L. (2001). Introduction to Distance Sampling: Estimating Abundance of Biological Populations.

[25] Buckland S.T., Anderson D.R., Burnham K.P., Laake J.L., Borchers D.L., Thomas L. (2004). Advanced Distance Sampling.

[26] Buckland S., Newman K.B., Fernández C., Thomas L., Harwood J. (2007). Embedding Population Dynamics Models in Inference. Stat. Sci..

[27] Anderson D.R., Burnham K.P., Laake J.L. (1993). Distance Sampling: Estimating Abundance of Biological Populations.

[28] Sprent P., Buckland S.T., Anderson D.R., Burnham K.P., Laake J.L. (1994). Distance sampling-estimating abundance of biological populations. J. Appl. Ecol..

[29] Le Moullec M., Pedersen Å.Ø., Yoccoz N., Aanes R., Tufto J., Hansen B. (2017). Ungulate population monitoring in an open tundra landscape: Distance sampling versus total counts. Wildl. Biol..

[30] Kent M. (2012). Vegetation Description and Data Analysis: A Practical Approach.

[31] Curtis J.T., McIntosh R.P. (1950). The interrelations of certain analytic and synthetic phytosociological characters. Ecology.

[32] Ijaz F. (2014). Biodiversity and Traditional Uses of Plants of Sarban Hills, Abbottabad. M.Phil. Thesis.

[33] Ijaz F., Rahman I.U., Iqbal Z., Alam J., Ali N., Khan S.M. (2018). Ethno-Ecology of the Healing Forests of Sarban Hills, Abbottabad, Pakistan: An Economic and Medicinal Appraisal.

[34] Nasir E., Ali S.I., Nasir E., Ali S.I. (1971). Papilionaceae. Flora West of Pakistan.

[35] Ali S.I., Nasir Y.J. (1989). Flora of Pakistan.

[36] Ali S.I., Qaiser M. (1995). Flora of Pakistan.

[37] Haq F., Ahmad H., Iqbal Z. (2015). Vegetation description and phytoclimatic gradients of subtropical forests of Nandiar Khuwar catchment District Battagram. Pak. J. Bot..

[38] Ravindranath N.H., Ostwald M. (2008). Carbon Inventory Methods Handbook for Greenhouse Gas Inventory, Carbon Mitigation and Roundwood Production Projects.

[39] McLean E.O. (1983). Soil pH and lime requirement. Methods Soil Anal. Part 2 Chem. Microbiol. Prop..

[40] Wilson M.J., Bayley S.E. (2012). Use of single versus multiple biotic communities as indicators of biological integrity in northern prairie wetlands. Ecol. Indic..

[41] Nelson D.W., Sommers L.E. (1996). Total Carbon, Organic Carbon, and Organic Matter. Methods of Soil Analysis: Part 3 Chemical Methods.

[42] Soltanpour P.N. (1991). Determination of Nutrient Availability and Elemental Toxicity by AB-DTPA Soil Test and ICPS. Advances in Soil Science.

[43] Rahman I.U., Afzal A., Iqbal Z., Ijaz F., Khan S.M., Khan S.A., Shah A.H., Khan K., Ali N. (2015). Influence of different nutrients application in nutrient deficient soil on growth and yield of onion. Bangladesh J. Bot..

[44] Rahman I.U., Ijaz F., Afzal A., Iqbal Z. (2017). Effect of foliar application of plant mineral nutrients on the growth and yield at-tributes of chickpea (*Cicer arietinum* L.) Under nutrient deficient soil conditions. Bangladesh J. Bot..

[45] Šmilauer P., Jan L. (2014). Multivariate Analysis of Ecological Data Using CANOCO.

[46] Mayor J.R., Sanders N.J., Classen A., Bardgett R.D., Clement J.-C., Fajardo A., Lavorel S., Sundqvist M.K., Bahn M., Chisholm C. (2017). Elevation alters ecosystem properties across temperate treelines globally. Nat. Cell Biol..

[47] Kenkel N.C. (2006). On selecting an appropriate multivariate analysis. Can. J. Plant Sci..

[48] McCune B., Mefford M. (2011). PC-ORD. Multivariate Analysis of Ecological Data. Version 6.

[49] R Core Team R (2013). A Language and Environment for Statistical Computing.

[50] Bano S., Khan S.M., Alam J., Alqarawi A.A., Abd_Allah E.F., Ahmad Z., Rahman I.U., Ahmad H., Aldubise A., Hashem A. (2018). Eco-Floristic studies of native plants of the Beer Hills along the Indus River in the districts Haripur and Abbottabad, Pakistan. Saudi J. Biol. Sci..

[51] Haq F., Ahmad H., Iqbal Z., Alam M., Aksoy A. (2017). Multivariate approach to the classification and ordination of the forest ecosystem of Nandiar valley western Himalayas. Ecol. Indic..

[52] Hill M.O. (1979). TWINSPAN: A FORTRAN Program for Arranging Multivariate Data in an Ordered Two-Way Table by Classification of the Individuals and Attributes.

[53] Terzi M., Bogdanović S., D’Amico F.S., Jasprica N. (2019). Rare plant communities of the Vis Archipelago (Croatia). Bot. Lett..

[54] Oksanen J., Blanchet F.G., Friendly M., Kindt R., Legendre P., McGlinn D., Minchin P.R., O’Hara R.B., Simpson G.L., Solymos P. (2007). The Vegan package. Community Ecol. Package.

[55] Fox J., Weisberg S. (2018). An R Companion to Applied Regression.

[56] Rahman I.U. (2020). Ecophysiological Plasticity and Ethnobotanical Studies in Manoor Area, Kaghan Valley, Pakistan. Ph.D. Thesis.

[57] Khan S.M. (2012). Plant Communities and Vegetation Ecosystem Services in the Naran Valley, Western Himalaya. Ph.D. Thesis.

[58] Hufkens K., Scheunders P., Ceulemans R. (2009). Ecotones in vegetation ecology: Methodologies and definitions revisited. Ecol. Res..

[59] Luo Z., Tang S., Li C., Fang H., Hu H., Yang J., Ding J., Jiang Z. (2012). Environmental Effects on Vertebrate Species Richness: Testing the Energy, Environmental Stability and Habitat Heterogeneity Hypotheses. PLoS ONE.

[60] Yang Z., Liu X., Zhou M., Ai D., Wang G., Wang Y., Chu C., Lundholm J.T. (2015). The effect of environmental heterogeneity on species richness depends on community position along the environmental gradient. Sci. Rep..

[61] Digby P.G.N., Kempton R.A. (2012). Multivariate Analysis of Ecological Communities.

[62] Biondi E., Feoli E., Zuccarello V. (2004). Modelling environmental responses of plant associations: A review of some critical concepts in vegetation study. Crit. Rev. Plant Sci..

[63] James F.C., McCulloch C.E. (1990). Multivariate analysis in ecology and systematics: Panacea or Pandora’s box?. Annu. Rev. Ecol. Syst..

[64] Beals M.L. (2006). Understanding community structure: A data-driven multivariate approach. Oecologia.

[65] Glatthorn J., Feldmann E., Tabaku V., Leuschner C., Meyer P. (2018). Classifying development stages of primeval European beech forests: Is clustering a useful tool?. BMC Ecol..

[66] Dufrêne M., Legendre P. (1997). Species assemblages and indicator species: The need for a flexible asymmetrical approach. Ecol. Monogr..

[67] Paudel P.K., Sipos J., Brodie J.F. (2018). Threatened species richness along a Himalayan elevational gradient: Quantifying the influ-ences of human population density, range size, and geometric constraints. BMC Ecol..

[68] Anderson M.J., Ellingsen K.E., McArdle B.H. (2006). Multivariate dispersion as a measure of beta diversity. Ecol. Lett..

[69] Haq F., Ahmad H., Iqbal Z. (2015). Vegetation composition and ecological gradients of subtropical-moist temperate Ecotonal forests of Nandiar Khuwar Catchment, Pakistan. Bangladesh J. Bot..

[70] Khan S.M., Harper D.M., Page S., Ahmad H. (2011). Species and community diversity of vascular flora along environmental gra-dient in Naran Valley: A multivariate approach through indicator species analysis. Pak. J. Bot..

[71] Klanderud K., Birks H.J.B. (2003). Recent increases in species richness and shifts in altitudinal distributions of Norwegian mountain plants. Holocene.

[72] Odland A., Birks H.J.B. (1999). The altitudinal gradient of vascular plant richness in Aurland, western Norway. Ecography (Cop.). Ecography.

[73] Weckström J., Korhola A. (2001). Patterns in the distribution, composition and diversity of diatom assemblages in relation to eco-climatic factors in Arctic Lapland. J. Biogeogr..

[74] Shaheen H., Ullah Z., Khan S.M., Harper D.M. (2012). Species composition and community structure of western Himalayan moist temperate forests in Kashmir. For. Ecol. Manag..

[75] Sahney S., Benton M.J., Ferry P.A. (2010). Links between global taxonomic diversity, ecological diversity and the expansion of ver-tebrates on land. Biol. Lett..

[76] Benton M.J. (2010). The origins of modern biodiversity on land. Philos. Trans. R. Soc. B Biol. Sci..

[77] Smith M., Facelli J., Cavagnaro T. (2018). Interactions between soil properties, soil microbes and plants in remnant-grassland and old-field areas: A reciprocal transplant approach. Plant Soil.

[78] Allen C.D., Breshears D.D. (1998). Drought-induced shift of a forest-woodland ecotone: Rapid landscape response to climate variation. Proc. Natl. Acad. Sci. USA.

[79] Crumley C.L. (1993). Analyzing historic ecotonal shifts. Ecol. Appl..

[80] Neilson R.P. (1993). Transient ecotone response to climatic change: Some conceptual and modelling approaches. Ecol. Appl..

