# Supplementary material for: Species Distribution Pattern and Their Contribution in Plant Community Assembly in Response to Ecological Gradients of the Ecotonal Zone in the Himalayan Region
Source: Plants (Basel). 2021 Nov 4;10(11):2372. doi: 10.3390/plants10112372 (PMC8623140; doi:10.3390/plants10112372)
Supplement: Supplementary file 1 [file plants-10-02372-s001.zip › plants-1357347-supplementary.pdf]

Table S1. List of plant species with their Importance values (IV) calculated based on vegetative characteristics of each sampling sites.

	Sampling sites						
**Species**	**S1**	**S2**	**S3**	**S4**	**S5**	**S6**	**S7**
Ach asp	2.74	0.57	2.46	0	0	0	0
Ach bid	0	1.38	0	0	0	0	0
Adi cap-ven	3.04	1.73	1.86	1.73	0	0	0
Adi ind	2.54	0	1.93	0	0	0	0
Ail alt	0	0	0	10.5	0.28	1.74	5.78
Ama vir	1.47	0	1.01	2.47	0	0	0
Ana arv	3.76	2.51	2.06	0	0	0	0
Arc min	3.64	2.52	2.78	0	0	0	0
Art abs	3.27	1.88	2.63	0	0	0	0.99
Ber cil	0	0	2.19	0	0	0	0
Ber lyc	0	4.54	8.43	0	0	0	0
Ber par	0	0	3.29	0	0	0	0
Bis amp	15.43	7.26	2.01	0	0	0	0
Bro dia	0	0	3.72	0	0	0	0
Bro sec	0	4.28	6.34	4.27	0	0	0
Bro tec	0	3.17	2.94	2.75	0	0	0
Bup nig	0	0	7.63	0	0	0	0
Can sat	0	0	0	2.3	0	0	0
Cap bur-pas	3.65	2.63	1.74	2.83	0	0	0
Car sp	3.76	0	0	0	0	0	0
Che alb	3.84	1.72	1.25	2.87	0	0	0.49
Cic int	2.76	0	0	0	0	0	0
Cir arv	4.26	0	2.78	0	0	0	0
Cle gra	7.36	5.38	2.73	0	0	0	0
Cli vul	3.73	2.47	3.54	4.36	0	0	0.73
Com ben	2.37	0	0	0	0	0	0
Con arv	0	0	2.01	3.65	0	0	0
Con jap	2.83	0.43	3.23	1.09	0	0	1.01
Cot acu	0	0	0	0	1.38	1.75	0
Cro sp	2.09	0	0	0	0	1.59	0
Cus ref	4.82	2.54	1.85	2.54	0	0	0
Cyn ape	0	0	0	2.47	0	0	0

Cyn dac	1.93	5.24	3.54	3.76	1.05	0.85	2.78
Cyn glo	3.87	2.06	3.18	4.83	0	0	0
Cyn mic	2.95	1.83	2.63	3.78	0	0	0
Cyp odo	0	0.76	0.38	0	0	0	0
Cyp rot	3.73	0	2.64	0	0	0	0
Dap muc	0	0	3.85	0	0	0	0
Dic bup	5.26	2.18	3.01	0	0	0	0
Dod vis	1.85	2.57	0	0	0	0	0
Duc ind	0	2.37	2.63	0	0	0	0
Dys amb	3.52	2.73	3.7	2.92	0	0	0
Eri can	4.02	2.31	0.56	0.46	0.76	0.18	2.97
Eup hel	3.27	2.84	3.06	2.04	0	0	0
Eup hir	2.71	0	2.64	0	0	0	0
Eup pro	2.31	2.01	0.67	1.9	0	0	0
Fic car	0	0	0	1.87	0	0	0
Fra hoo	0	0	0	1.04	0	0	0
Fra nub	4.02	3.72	0	0	0	0	0
Fra xan	0	0	3.58	0	0	0	0
Fum ind	2.16	3.51	2.85	2.87	0	0	0
Gal apa	0	3.42	2.61	0	0	1.84	0
Ger nep	4.26	0	0	0	0	0	0
Ger wal	5.27	0	0	0	0	0	0
Imp bic	0	6.27	3.24	0	0	0	0
Ind heb	10.62	3.25	5.58	0	0	0	0
Ind het	17.25	8.17	10.32	13.63	19.88	8.04	16.53
Ipo nil	0	0	2.95	0	0	0	0
Iso rug	1.01	4.28	2.4	5.86	0.67	2.06	0
Jas hum	0	5.38	0	0	0	0	0
Jug reg	0	0	5.63	0	0	0	0
Lau pro	0	0	0	0	0	0	0.76
Lep chi	0	0	0	0	25.72	19.73	18.65
Lep vir	0	0	0	2.58	4.21	0.32	0.72
Lin sp	0	0	4.73	0	0	0	0
Mal cor	0	0	1.37	0	0	0	0
Mal par	0.85	0	0	0	0	0	0.47
Mal neg	2.01	0	0	0	0	0	0.73
Med sat	3.05	0	3.1	0	0.19	0	0.84

Mic bif	0	0	0	5.78	6.85	10.67	0
Oen ros	3.78	0	0	0	0	0	0
Oxa cor	2.13	1.08	0.54	0.86	0	0	0.78
Par hys	0	0	0	4.63	0	0	0
Par jac	13.26	9.66	4.63	8.64	0	0	0
Pen ori	0	0	3.07	0	0	0	0
Per cap	2.06	1.85	2	1.85	0	0	0
Pim ste	7.34	2.85	0	2.85	0	0	0
Pla lan	0	0	0	0	0.21	0	1.04
Pla maj	0	0	3.06	0	0	0	0
Poa ann	0	4.23	2.53	2.23	0	0	0
Poa inf	0	6.26	2.38	3.17	0	0	0
Que inc	0	0	2.1	0	20.1	12.5	14.7
Pyr pas	0	4.36	3.52	4.01	0	0	0
Rum den	0	0	2.84	0	0	0	0
Rum nep	0	0	3.32	0	0	0	0
Sal alb	0	0	1.85	0	0	0	0
Sil con	0	0	3.64	0	0	0	0
Sol nig	0	0	0	0	0	0	1.84
Sol sur	0	0	0	0	0.84	0	1.46
Son asp	0	0	0	0	0.18	0	0.87
Sor hal	0	0	0	0	0	0	1.72
Sorb tom	0	2.86	5.64	2.86	0	0	1.93
Tar cam	0	0	2.64	0	0	0	0
Tri rep	1.05	1.73	2.75	1.73	0	0	0.58
Uro pan	0	4.02	5.62	4.02	0	0	0
Ver tha	0	0	0	0	0.11	0	0.93
Ziz sp	0	0	0	0	31	44.45	13.04

Table S2. Means of ecological gradients measured at three different transects of each sampling site were recorded (wind speed averages are given as integers).

Ecological gradients	Sampling sites						
	**S1**	**S2**	**S3**	**S4**	**S5**	**S6**	**S7**
Altitude	1891.5	1896.3	1886.0	1789.6	1780.8	1789.3	1787.2
Latitude	34.7033	34.70323	34.70326	34.69885	34.69549	34.69564	34.69558
Longitude	73.61694	73.61694	73.61723	73.60368	73.59976	73.59962	73.59954
Slope Angle	75	80	80	45	70	65	55
Temp	29	29.2	27.8	26.8	31.8	28.8	29.4
Humidity	57.2	60.3	63.4	63.1	51.1	56	56.9
Heat index	29.8	30.4	33.3	26.9	33.2	29.8	34.3
Wind speed	1.5	0	1.5	1	1	2	3
Dew point	20.1	20.3	21.4	18.6	19.8	18.9	21
Wet bulb	22.6	22.2	23.8	20.8	22.9	22.2	23.3
Baro Press	805.2	804.7	805.8	815.6	816.4	815.6	815.6
Alt dens	9545	9474	9430	8950	9465	9209	9247
pH	6.5	6.5	6.4	6.6	6	5.9	5.9
EC	0.78	1.09	1.75	1.54	4.14	5.4	2.82
OM	0.85	1.15	1.37	0.7	1.08	1.24	1.25
CaCO3	5.6	7.3	6.9	2.8	7.6	5.8	5.8
K	210	215	220	200	208	220	220
P	11	9	13	9.2	12	15	4.1
Sand	44	50	50	26.4	45.6	29.2	52
Silt	36	34	36	49.4	28.2	56.2	31
Clay	20	16	14	24.2	26.2	14.6	17
